# Joint effects of arterial stiffness and polygenic risk on kidney function: the Dongfeng-Tongji cohort

**DOI:** 10.3389/fcvm.2026.1717046

**Published:** 2026-03-13

**Authors:** Haiqing Zhang, Rui Zhang, Yaling He, Xuefeng Lai, Rong Peng, Miao Liu, Biao Zhang, Huihui Wang, Xingjie Hao, Liangle Yang, Xiaomin Zhang

**Affiliations:** 1 Department of Occupational and Environmental Health, Ministry of Education Key Laboratory of Environment and Health, and State Key Laboratory of Environmental Health (Incubating), School of Public Health, School of Tongji Medical College, Huazhong University of Science and Technology, Wuhan, Hubei, China; 2 Henan Provincial Modern Hospital Management Research Center, Henan Provincial People’s Hospital, People’s Hospital of Zhengzhou University, Zhengzhou, Henan, China; 3 Department of Epidemiology and Biostatistics, School of Public Health, School of Tongji Medical College, Huazhong University of Science and Technology, Wuhan, Hubei, China

**Keywords:** arterial stiffness, interaction, joint association, kidney function, polygenic risk score

## Abstract

**Objective:**

Arterial stiffness may contribute to decline in kidney function, but it is unknown whether the association of arterial stiffness with kidney function might be influenced by genetic risk. We aimed to investigate the association of arterial stiffness evaluated by brachial-ankle pulse wave velocity (baPWV) and polygenic score (PRS) with kidney function and the interaction between baPWV and PRS in middle-aged and older Chinese adults.

**Methods:**

We included 13,494 participants with an average age of 68 years from the Dongfeng-Tongji cohort in 2018. A PRS was calculated using 67 variants associated with estimated glomerular filtration rate (eGFR).

**Results:**

A total of 1,939 (14.4%) participants were defined as chronic kidney disease (CKD) (eGFR <60 mL/min/1.73 m^2^). Increased baPWV or PRS was dose-dependently associated with lower eGFR or higher risk of CKD. Compared to the participants with baPWV <1,400 cm/s, the *β* coefficient of eGFR was −1.755 (95% CI, −2.716, −0.794) and odds ratio (OR) of CKD was 1.39 (95% CI, 1.15–1.69) in participants with baPWV ≥1,800 cm/s, with stronger associations among those with PRS in the top quintile than the bottom quintile. The combination of baPWV ≥1,800 cm/s and the top quintile of PRS was associated with decreased eGFR (β, −5.317; 95% CI, −7.161, −3.472) and increased odds of CKD (OR, 1.74; 95% CI, 1.19–2.55). No interaction between baPWV and PRS on kidney function was found.

**Conclusions:**

BaPWV was associated with both reduced eGFR and elevated CKD risk, especially in the participants with high genetic risk.

## Introduction

Chronic kidney disease (CKD) poses a major global health burden, affecting around 9.1% of the world's population in 2017 (1). The disease accounted for over 1.2 million deaths, marking a 41.4% increase in mortality rates compared to 1999 (1). In China, approximately 82 million adults had CKD in 2019 (2). A better understanding of CKD etiology and improved prevention strategies are critically urgent worldwide to minimize its burden.

Increased arterial stiffness is detectable in the early stages of CKD, as it can induce excessive pulsatility in renal microvascular blood flow, ultimately leading to a diminished estimated glomerular filtration rate (eGFR) (3). Existing epidemiological studies explored an independent association between arterial stiffness and kidney function, but the results remained inconsistent. Several studies showed that arterial stiffness, indicated by increased pulse wave velocity (PWV), was associated with decreased kidney function (4) or an increased CKD risk (5–7), whereas other studies failed to establish a positive association (8, 9). Those studies mainly focused on high-risk patients with comorbid conditions such as hypertension and CKD, limiting generalizability. Two prior studies focused on the general population, finding that PWV was linked with the risk of CKD in Chinese adults (10, 11). Consequently, the relationship between baPWV and kidney function remains to be fully clarified.

Moreover, it is widely acknowledged that genetic factors significantly contribute to the pathogenesis of CKD. Recent genome-wide association study (GWAS) identified many genetic variants associated with kidney-related traits including creatinine, eGFR, and blood urea nitrogen (BUN) (12), with heritability estimates of CKD ranging from 25% to 44% (13). The polygenic risk score (PRS) quantifies the common-variant risk posed by a specific disease or trait and is calculated by summing the effects of various single nucleotide polymorphisms (SNPs). Previously, the Rotterdam Study included 3,666 older adults aged 65 years and calculated PWV-related PRS, finding no significant association of genetic susceptibility with eGFR decline or incident CKD (4). However, the interplay arterial stiffness and kidney-related PRS on kidney function is still not well understood. Elucidating these associations could provide novel insights into CKD etiology.

Based on the Dongfeng-Tongji cohort, we thus aimed to (1) explore the independent association of brachial-ankle PWV (baPWV) with kidney function including eGFR, BUN, and CKD, (2) investigate whether this relationship could be modified by PRS for kidney function, and (3) assess the joint associations of baPWV and PRS with kidney function.

## Methods

### Study population

The current study utilized data from the Dongfeng-Tongji cohort, which was previously described in detail (14, 15). Briefly, a total of 27,009 retired employees from Dongfeng Motor Corporation were included in the baseline survey including questionnaires, medical check-up, and blood collection in Shiyan city, Hubei province of China from September 2008 to June 2010. Follow-up surveys were performed every 5 years, with new participants included in the survey of 2013 and 2018. Participants received the repeated baseline questionnaires, physical examinations, and bio-sample collection in the follow-up examination. Of 36,215 participants in 2018, a total of 21,801 subjects with kidney function also completed measurement of arterial stiffness. After excluding 8,307 individuals with missing genotype data, 13,494 participants remained for analysis (Supplementary Figure S1).

The study was approved by the Ethics Committee of Tongji Medical College, Huazhong University of Science and Technology and have been performed in accordance with the ethical standards as laid down in the 1964 Declaration of Helsinki and its later amendments or comparable ethical standards. Written informed consent was obtained from all participants.

### Assessments of arterial stiffness

The baPWV was measured using an oscillometric device (BP−203RPEIII; Omron, Kyoto, Japan) as described previously (16). Participants were asked to lie in supine position for at least 5 to 10 min during the measurement. Simultaneously, the device recorded bilateral bra and tibial arterial waveforms, lead I electrocardiogram, and phonocardiogram. Occlusion cuffs equipped with plethysmographic and oscillometric sensors were applied to the arms and ankles to measure and pulse waves. The automatic calculation of baPWV was utilized the following formula:

baPWV = (Distance from heart to ankle—Distance from heart to brachium)/Time difference between brachium and ankle arterial pressure waves.

The mean value of left and right baPWV was used in the final analysis. Previous studies reported that baPWV value with a cutoff of 1,400 or 1,800 cm/s is widely used as an indicator of cardiovascular risk (17, 18). In order to comprehensively evaluate the effects of different baPWV group, we thus categorized the participants into three groups based on their baPWV measurements: <1,400 cm/s, 1,400–<1,800 cm/s, and ≥1,800 cm/s.

### Assessment of kidney function

The outcome in this study focused on kidney function including BUN, eGFR, and CKD. Serum creatinine and BUN concentrations were determined by an Abbott Architect Ci8200 automatic analyzer (Abbott Laboratories. Abbott Park, Illinois, USA) according to standard procedures and reagents specified by the manufacturer. The calculation of eGFR was performed using the Chronic Kidney Disease Epidemiology Collaboration equation (19):

$$\mathrm{eGFR}=141\times \min(\mathrm{creatinine}/k,1{)}^{\alpha }\times \max(\mathrm{creatinine}/k,\;1{)}^{-1.209}\times 0.993^{\mathrm{Age}}\times (1.018\;\mathrm{if}\;\mathrm{female})$$

where k is 0.7 for females and 0.9 for males, α is −0.329 for females and −0.411 for males, min indicates the minimum of creatinine/k or 1, and max indicates the maximum of creatinine/k or 1. We defined CKD as an eGFR <60 mL/min/1.73 m^2^.

### Genotyping and calculation of PRS for CKD

Participants of our study were genotyped using the Illumina Infinium OminZhongHua-8 array. In quality control procedures, the dataset was phased and imputed to an East Asian GRCh38 reference panel with 3,931 East Asians from 1,000 Genomes Project phase 3 (1KGP3) (20) and SG10K (21) using Eagle (v2.4.1) (22) and Minimac4 (23). We further excluded variants with minor allele frequency <0.001 or *R*^2^ < 0.3 for downstream analyses.

We created an eGFR-based PRS using 67 SNPs (excluding 2 variants at sex chromosome) associated with eGFR (*P* < 5 × 10^−8^) based on the previous GWASs in East Asian ancestry (Supplementary Table S1) (12). Individual SNP was recoded as 0, 1, and 2 according to the number of risk alleles; and then multiplied by the effect size (β-coefficient) on eGFR to calculate the PRS: PRS = (β_1_ × SNP_1_ + β_2_ × SNP_2_+ … +β_67_ × SNP_67_) × (67/sum of the β coefficients). We defined the allele associated with decreased eGFR, increased creatinine and BUN as risk allele. The effect size (β-coefficient) for each SNP was obtained from the previous genome-wide association study (GWAS) in 162,255 participants of East Asian ancestry (12). A higher PRS is predicted to have lower eGFR, higher creatinine, and higher BUN, presenting higher risk of CKD. The participants were categorized into low (the bottom quintile), medium (the second to the fourth quintile), and high (the top quintile) according to PRS.

### Assessment of covariates

Socio-demographic, lifestyle, and medical history data were collected via semi-structured questionnaires administered by trained investigators. We classified education as primary school or below, junior or senior high school, and college or above. Marital status was divided into married/remarried, and unmarried/divorced/widowed.

Systolic blood pressure (SBP) and diastolic blood pressure (DBP) were measured and recorded by a well-trained nurse or physician. Body mass index (BMI) was calculated as weight (kg) divided by height (m) squared. Fasting blood was collected for laboratory assays of fasting glucose, triglycerides (TG), total cholesterol (TC), high density lipoprotein cholesterol (HLD-C), low density lipoprotein cholesterol (LDL-C).

### Statistical analysis

Participant characteristics were presented as mean ± SD for continuous variables and frequency (percentages) for categorical variables according to baPWV or PRS. Chi-square test was used to compare categorical variables, while analysis of variance was used for continuous variables. Restricted cubic splines with three knots at the fifth, 50th, and 95th percentiles were generated to visually examine the association between baPWV and eGFR, BUN, and CKD. The general linear regression was employed to assess the associations of baPWV with eGFR and BUN. Adjustments were made for various covariates including age, gender, marital status, education, smoking status, drinking status, physical activity, BMI, fasting plasma glucose, cardiovascular disease, cancer, SBP, TG, TC, and HDL-C. Logistic regression models were fitted to estimate the odds ratios (ORs) and 95% confidence intervals (CIs) for the association of baPWV with CKD, adjusting for the same covariates in the aforementioned models. Additionally, baPWV were analyzed continuously to assess linear trends. We investigated the relationship between PRS and kidney function, adjusting for age, gender, and the first ten principal components.

Stratified analyses were conducted according to PRS to explore potential modification effects. Interaction was evaluated by including the interaction term between PRS and baPWV in the models. We also visualized the interaction effects of PRS and baPWV on kidney function using R package “interplot”. We further estimated the joint associations of PRS and baPWV with kidney function using the group of low PRS and baPWV <1,400 cm/s as the reference. Additive interaction for CKD was assessed by using the relative excess risk due to the interaction (RERI) and the attributable proportion due to the interaction (AP) (24). A 95% CI including zero indicated no additive interaction.

To evaluate the robustness of our results, we conducted several sensitivity analyses. We repeatedly performed the primary analyses by excluding individuals with eGFR <15 mL/min/1.73 m^2^, who have potential severe kidney failure. We further restricted the sample to participants without cancer or cardiovascular disease, which is an important risk factor of CKD. Finally, we repeated the analyses based on the eGFR using Modification of Diet in Renal Disease equation (19). All statistical analyses were performed with SAS (SAS Institute, Cary, NC, USA, version 9.4), or R (version 4.1.2). Two-sided *P* values of less than 0.05 were considered as statistically significant.

## Results

### Participant characteristics

Table 1 summarized participant characteristics according to baPWV. Of the 13,494 participants, the mean (SD) age was 68.3 (8.0) years, and 7,975 (59.1%) were female. A total of 1,939 (14.4%) participants were defined as CKD. Compared to participants with baPWV <1,400 cm/s, those with baPWV ≥1,800 cm/s were likely to be older, mainly female, drinker, engage in less exercise, and have a poorer kidney function (higher creatinine, lower eGFR, higher BUN, and higher prevalence of CKD). In addition, participants with top PRS quintile (high) had increased creatinine, decreased eGFR, higher BUN, and prevalence of CKD in comparison to participants with the bottom PRS quintile (low) (Supplementary Table S2).

**Table 1:** Participant characteristics of study population by baPWV.

Characteristics	Total	baPWV (cm/s)			
		<1,400	1,400–<1,800	≥1,800	*P*
No. of participants, *n* (%)	13,494 (100.0)	2,167 (16.1)	5,776 (42.8)	5,551 (41.1)	
Age (year), mean ± SD	68.3 ± 8.0	62.0 ± 7.3	67.2 ± 7.3	71.9 ± 7.0	<0.001
Gender, *n* (%)					<0.001
Male	5,519 (40.9)	512 (23.6)	2,326 (40.3)	2,681 (48.3)	
Female	7,975 (59.1)	1,655 (76.4)	3,450 (59.7)	2,870 (51.7)	
Education, *n* (%)					<0.001
Primary school or below	3,101 (23.0)	286 (13.2)	1,208 (20.9)	1,607 (28.9)	
Junior or senior high school	9,240 (68.5)	1,684 (77.7)	4,101 (71.0)	3,455 (62.2)	
College or above	1,153 (8.5)	197 (9.1)	467 (8.1)	489 (8.8)	
Marital status, *n* (%)					<0.001
Unmarried/divorced/widowed	1,905 (14.1)	257 (11.9)	736 (12.7)	912 (16.4)	
Married/remarried	1,1,588 (85.9)	1,910 (88.1)	5,039 (87.3)	4,639 (83.6)	
Current smoker, *n* (%)	1,545 (11.5)	212 (9.8)	712 (12.3)	621 (11.2)	0.005
Current drinker, *n* (%)	2,535 (18.8)	353 (16.3)	1,051 (18.2)	1,131 (20.4)	<0.001
Physical activity, *n* (%)	12,350 (91.5)	1,980 (91.4)	5,328 (92.3)	5,042 (90.8)	0.025
BMI (kg/m^2^), mean ± SD	24.7 ± 3.4	24.0 ± 3.4	24.7 ± 3.4	25.0 ± 3.4	<0.001
SBP (mmHg), mean ± SD	138.8 ± 21.1	121.3 ± 16.5	135.6 ± 18.0	149.0 ± 20.2	<0.001
FPG (mmol/L), mean ± SD	6.0 ± 1.9	5.4 ± 1.1	5.8 ± 1.6	6.3 ± 2.3	<0.001
TG (mmol/L), mean ± SD	1.5 ± 1.0	1.3 ± 0.8	1.4 ± 0.9	1.5 ± 1.2	<0.001
TC (mmol/L), mean ± SD	4.8 ± 1.0	4.9 ± 1.0	4.8 ± 1.0	4.7 ± 1.1	<0.001
HDL-C (mmol/L), mean ± SD	1.4 ± 0.4	1.5 ± 0.4	1.4 ± 0.4	1.4 ± 0.4	<0.001
Cr (μmol/L), mean ± SD	76.0 ± 26.4	70.0 ± 18.1	75.1 ± 25.4	79.3 ± 29.6	<0.001
eGFR (mL/min/1.73 m^2^), mean ± SD	79.8 ± 16.8	85.6 ± 15.9	81.0 ± 16.4	76.4 ± 16.8	<0.001
BUN (mmol/L), mean ± SD	5.5 ± 1.6	5.2 ± 1.5	5.5 ± 1.6	5.6 ± 1.7	<0.001
CKD, *n* (%)	1,939 (14.4)	196 (9.0)	719 (12.4)	1,024 (18.4)	<0.001

baPWV, brachial-ankle pulse wave velocity; BMI, body mass index; BUN, blood urea nitrogen; CKD, chronic kidney disease; Cr, creatinine; eGFR, estimated glomerular filtration; FPG, fasting plasma glucose; HDL-C, high density lipoprotein cholesterol; SBP, systolic blood pressure; SD, standard deviation; TC, total cholesterol, and TG, triglyceride.

### Association of baPWV with kidney function

We found a dose-response association between increased baPWV and decreased eGFR (*P*_Overall_ < 0.001; *P*_Non−linear_ = 0.658; Supplementary Figure S2). Each 100 cm/s increase in baPWV was associated with reduced eGFR (β, −0.212; 95% CI, −0.300, −0.123; *P*_trend_ < 0.001). After dividing baPWV into three groups, participants with baPWV ≥1,800 cm/s had an adjusted β (95% CI) of −1.755 (−2.716, −0.794) for eGFR compared to participants with bwPWV <1,400 cm/s (Table 2). Likewise, increased baPWV was related to higher odds of CKD in a dose-dependent fashion (*P*_Overall_ < 0.001; *P*_Non−linear_ = 0.225; Supplementary Figure S2). Those with baPWV ≥1,800 cm/s had a 39% greater odds for CKD (Table 2). No significant association between baPWV and BUN was observed.

**Table 2:** The relationship between baPWV and kidney function.

Models	baPWV (cm/s)				
	<1,400	1,400–<1,800	≥1,800	*P*_trend_	Per 100 cm/s increase in baPWV
β (95% CI) of eGFR					
Model 1	0 (Ref)	**−0.479** **(−1.264, 0.307)**	**−1.333** **(−2.178, −0.487)**	<0.001	**−0.135** **(−0.211, −0.059)**
Model 2	0 (Ref)	**−0.511** **(−1.344, 0.321)**	**−1.755** **(−2.716, −0.794)**	<0.001	**−0.212** **(−0.300, −0.123)**
β (95% CI) of BUN					
Model 1	0 (Ref)	0.024 (−0.057, 0.106)	0.026 (−0.061, 0.114)	0.893	−0.001 (−0.008, 0.007)
Model 2	0 (Ref)	0.053 (−0.033, 0.139)	0.083 (−0.016, 0.183)	0.401	0.004 (−0.005, 0.013)
OR (95% CI) of CKD					
Model 1	1 (Ref)	**1.07** **(0.90–1.27)**	**1.32** **(1.11–1.57)**	<0.001	**1.03** **(1.01–1.04)**
Model 2	1 (Ref)	**1.08** **(0.90–1.29)**	**1.39** **(1.15–1.69)**	<0.001	**1.04** **(1.02–1.05)**

Model 1: Adjusted for age and gender.

Model 2: Adjusted for age, gender, marital status, education, smoking status, drinking status, physical activity, body mass index, fasting plasma glucose, cardiovascular disease, cancer, systolic blood pressure, triglyceride, total cholesterol, and high density lipoprotein cholesterol.

Bold values denote statistically significant.

baPWV, brachial-ankle pulse wave velocity; BUN, blood urea nitrogen; CI, confidence interval; CKD, chronic kidney disease; eGFR, estimated glomerular filtration; OR, odds ratio.

### Association of PRS with kidney function

We further observed that PRS was significantly with decreased eGFR, elevated BUN, and higher CKD (Table 3). Supplementary Figure S3 showed that elevated PRS was related to decreased eGFR (*P*_Overall_ < 0.001; *P*_Non−linear_ = 0.283), increased BUN (*P*_Overall_ < 0.001; *P*_Non−linear_ = 0.25), and higher CKD prevalence (*P*_Overall_ < 0.001; *P*_Non−linear_ = 0.246) in a dose-dependent fashion. Model 1 showed that each one standard deviation increase in PRS was linked to a decrease of 1.495 in eGFR, an increase of 0.124 in BUN, and a 14% increase in CKD (Table 3). The results remained consistent even after adjusting for age, gender, and the first ten principal components. The fully-adjusted β (95% CI) of eGFR and BUN was −1.500 (−1.780, −1.220) and 0.118 (0.089, 0.147), respectively, for each SD increment of PRS. The OR for CKD was 1.15 (95% CI, 1.09–1.21) in the group of top PRS quintile (high) compared with the group of bottom PRS quintile (low). Using continuous PRS, linear association for CKD was observed (*P*_trend_ < 0.001, Table 3). We also found no significant association between PRS and baPWV (data not shown).

**Table 3:** The association of PRS with kidney function.

Models	PRS				
	Low	Medium	High	*P*_trend_	Per one SD increase in PRS
β (95% CI) of eGFR					
Model 1	0 (ref)	**−1.580** **(−2.250, −0.909)**	**−3.878** **(−4.699, −3.057)**	<0.001	**−1.495** **(−1.755, −1.236)**
Model 2	0 (ref)	**−1.430** **(−2.156, −0.705)**	**−3.941** **(−4.832, −3.051)**	<0.001	**−1.500** **(−1.780, −1.220)**
β (95% CI) of BUN					
Model 1	0 (ref)	**0.169** **(0.100, 0.239)**	**0.326** **(0.241, 0.412)**	<0.001	**0.124** **(0.097, 0.151)**
Model 2	0 (ref)	**0.149** **(0.073, 0.225)**	**0.304** **(0.211, 0.397)**	<0.001	**0.118** **(0.089, 0.147)**
OR (95% CI) of CKD					
Cases/N	354/2,698	1,129/8,097	456/2,699		
Model 1	1 (ref)	1.08 (0.95–1.23)	**1.36** **(1.16–1.58)**	<0.001	**1.14** **(1.09–1.20)**
Model 2	1 (ref)	1.03 (0.90–1.19)	**1.36** **(1.16–1.60)**	<0.001	**1.15** **(1.09–1.21)**

Model 1: Adjusted for age and gender.

Model 2: Adjusted for age, gender, and first ten principal components.

The PRS was classified as low (the bottom quintile), medium (second to the fourth quintile), and high (the top quintile).

Bold values denote statistically significant.

baPWV, brachial-ankle pulse wave velocity; BUN, blood urea nitrogen; CI, confidence interval; CKD, chronic kidney disease; eGFR, estimated glomerular filtration; OR, odds ratio; PRS, polygenetic score; SD, standard deviation.

### Association of baPWV with kidney function stratified by PRS

**Figure 1:**
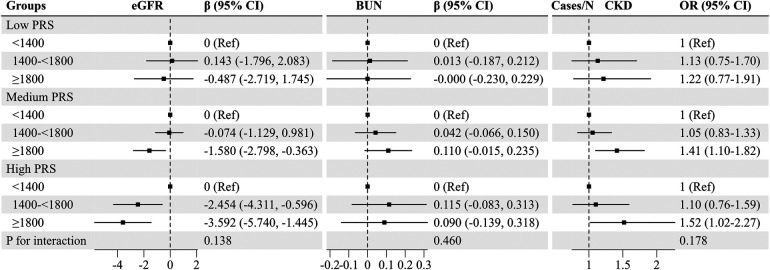
Associations of baPWV with kidney function stratified by PRS. Adjusted for age, gender, marital status, education, smoking status, drinking status, physical activity, body mass index, fasting plasma glucose, cardiovascular disease, cancer, systolic blood pressure, triglyceride, total cholesterol, and high density lipoprotein cholesterol. The PRS was classified as low (the bottom quintile), medium (the second to the fourth quintile), and high (the top quintile). baPWV, brachial-ankle pulse wave velocity; BUN, blood urea nitrogen; CI, confidence interval; CKD, chronic kidney disease; eGFR, estimated glomerular filtration; OR, odds ratio; PRS, polygenetic score.

We further evaluated the association of baPWV with kidney function stratified by PRS (Figure 1). Despite the non-significant interaction between baPWV and PRS for eGFR (*P* for interaction = 0.138), higher baPWV was associated with a more pronounced reduction in eGFR among individuals with higher PRS. The β (95% CI) of eGFR in the group of baPWV ≥1800 cm/s vs. the group of baPWV <1,400 cm/s was −3.592 (−5.740, −1.445) among participants with high PRS, −1.580 (−2.798, −0.363) among participants with medium PRS, and −0.487 (−2.719, 1.745) among those with low PRS (Figure 1). Similar results were found for CKD, but not for BUN. Furthermore, interactive plots also illustrated non-significant interactions for eGFR, BUN, and CKD (Supplementary Figure S4).

### Joint associations of PRS and baPWV with kidney function

**Figure 2:**
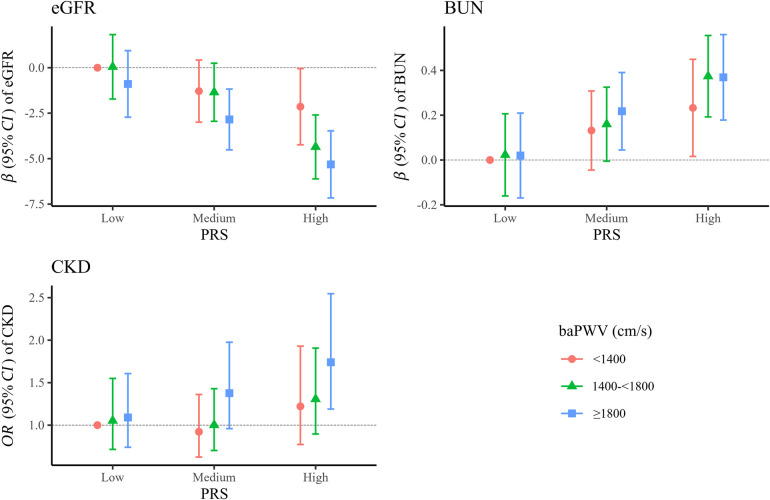
Joint association of PRS and baPWV with kidney function. Adjusted for age, gender, marital status, education, smoking status, drinking status, physical activity, body mass index, fasting plasma glucose, cardiovascular disease, cancer, systolic blood pressure, triglyceride, total cholesterol, and high density lipoprotein cholesterol. The PRS was classified as low (the bottom quintile), medium (second to the fourth quintile), and high (the top quintile). baPWV, brachial-ankle pulse wave velocity; BUN, blood urea nitrogen; CKD, chronic kidney disease; CI, confidence interval; eGFR, estimated glomerular filtration; OR, odds ratio; PRS, polygenetic score.

We observed joint associations of PRS and baPWV with kidney function (Figure 2 and Supplementary Table S3). Participants with high PRS and baPWV ≥1,800 cm/s had the lowest eGFR (β, −5.317; 95% CI, −7.161, −3.472) and the highest odds of CKD (OR, 1.74; 95% CI, 1.19–2.55) than those with low PRS and baPWV <1,400 cm/s. No significant additive interactions between baPWV and PRS for CKD were found (data not shown).

### Sensitivity analysis

Sensitivity analyses results were shown in Supplementary Tables S4, S5. The association between baPWV and kidney function was robust and stable throughout all the sensitivity analyses. The joint associations yielded consistent results, showing an even more prominent effect of baPWV on individuals with high PRS.

## Discussion and conclusion

This study is the first to investigate whether PRS could modify the associations between baPWV and kidney function. We found that elevated baPWV were significantly associated with decreased eGFR and increased odds of CKD in a dose-response fashion, but not with BUN. Notably, the strength of the association was greater among participants in the top PRS quintile compared to those in the bottom. Participants with high PRS and baPWV ≥1,800 cm/s had the lowest eGFR and highest CKD risk.

Previous studies on the association of baPWV with kidney function focused on chronic disease participants (i.e., hypertension and CKD) with conflicting results (5–7). Townsend et al. (6) demonstrated that higher PWV associated with CKD progression or death in 2,795 CKD patients. A Japanese study involving 184 patients with essential hypertension found that PWV increased more in severe CKD than in mild (5). Similarly, a smaller Chinese study of 547 hospitalized hypertensive patients found that carotid-femoral PWV was significantly elevated in patients with CKD (7). Conversely, two prior studies did not observe significant association of carotid-femoral PWV with lower eGFR when subjects were restricted to those with hypertension (*N* = 592) (8) or CKD (*N* = 913) (9). A relatively large longitudinal study of 10,535 individuals in China indicated that baPWV was predictive of a higher risk of CKD (11). In addition, results from another longitudinal study (*N* = 7,154) suggested that carotid-femoral PWV was linked with incident CKD but not with reduced eGFR in the general Chinese population (10). The very small number of participants with reduced eGFR (*N* = 68) might have lacked the statistical power to yield a significant association with eGFR. Furthermore, the results might be overestimated due to the broader definitions of CKD, which considered both decreased eGFR (<60 mL/min/1.73 m^2^) or the presence of proteinuria. Our study extended the work with a larger sample size (*N* = 13,494) and found that elevated baPWV was related to a 0.212 mL/min/1.73 m^2^ decrease in eGFR and a 4% increased CKD (defined as eGFR <60 mL/min/1.73 m^2^) in the Chinese middle-aged and older adult. This finding suggests that interventions targeted at reducing arterial stiffness may have the potential to lower the risk of CKD, although this hypothesis requires confirmation in future prospective studies and randomized controlled trials.

In addition, our study revealed no association between baPWV and BUN. We incorporated BUN as an additional marker to evaluate kidney function comprehensively, together with eGFR and CKD. However, BUN is influenced by multiple extra-renal factors such as protein intake, catabolic state, hydration, and medications, which increase its variability and may obscure associations with exposures like arterial stiffness (25). This probably explains the lack of a significant baPWV-BUN association in our study. In contrast, eGFR—derived from the standard equation—constitutes a more precise and validated measure of kidney function, while CKD, defined by an eGFR <60 mL/min/1.73 m², represents a clinically robust endpoint. Therefore, the primary finding of our study is the significant, dose-dependent association between baPWV and eGFR, which serves as a more reliable indicator of kidney function. Our findings showed that subjects with high PRS had higher CKD risk than those with low PRS. Sedaghat et al. (4) calculated a weighted PRS comprising nine PWV-related SNPs and showed no association of PWV-related PRS with kidney function in the Netherlands. Another study conducted in Australia and the United States revealed that PRS for eGFR are linked to CKD risk among individuals aged 70 and older, underscoring the persistent influence of genetic factors in kidney function even in later life. These results imply that PRS may be a useful tool for predicting CKD risk in elderly (26). Furthermore, Khan and colleagues constructed a PRS for CKD that encapsulates the polygenic influences on renal function identified through recent GWAS involving 15 independent cohorts representing four ancestral populations (78% European), with their data showing that individuals in the top 2% of the risk score distribution exhibited a nearly threefold elevation in CKD risk (27). However, performance of PRS can differ across ancestries due to genetic differences (28). In the current study, we first developed a PRS for kidney function based on East Asian GWAS data, independently linking a higher PRS to increased CKD risk in the Chinese population.

Previous studies demonstrated interactions between individual PRS of eGFR and environmental factors on kidney function (29–31). For the first time, we explored the interaction of PRS with baPWV regarding kidney function, finding no significant interaction but that the association was more pronounced among those in the top PRS quintile than those in the bottom. This could be explained by the fact that increase in arterial stiffness evaluated by baPWV might precede the decline in eGFR (11). Our study had limited power to detect interaction, and larger prospective studies are needed to confirm these patterns. Furthermore, the current study also showed that high PRS in combination with increased baPWV were jointly associated with the elevated odds of impaired kidney function. A significant joint association between baPWV and genetic susceptibility for CKD were observed, indicating a combined effect larger than individual effects (32). This supports the potential utility of interventions aimed at reducing arterial stiffness as a strategy for CKD prevention, especially in populations with a high PRS. In the stratified or joint analyses, we did not adjust for principal components to avoid overfitting the model with redundant covariates included. This might have the minimal impact on the results, as indicated by the association of the PRS with kidney function. Large-scale prospective studies in diverse populations are needed for confirmation. Our findings offer new insights into gene-environment mechanisms in CKD etiology.

Several mechanisms may underlie the role of arterial stiffness in kidney deterioration. Arterial stiffening, especially in older adults, could lead to the transmission of excessive pulsatile pressures and flowed into renal microvasculature, potentially causing microvascular damage and reductions in kidney function (33). In addition, previous studies suggested an association of chronic viral infections with increased arterial stiffness (34, 35). Therefore, increased risk of atheroma might be a consequence of chronic infection, which could cause the decline in kidney function, as chronic infection is also a feature of CKD (36). Our results could enhance understanding of pathophysiological pathways linking arterial health and kidney function from both genetic and environmental perspectives.

Notable strengths of this study include a large sample size for the arterial stiffness and genotype information. In addition, we derived a PRS from extensive East Asian GWAS. Furthermore, we systematically investigated the existence of interactions between PRS and baPWV on CKD from multiple aspects.

However, there are certain limitations to consider. Firstly, the cross-sectional design restricts causal inference, necessitating future prospective cohort studies for robustness. Secondly, baPWV is not considered the gold-standard for measuring arterial stiffness, but it closely correlates with carotid-femoral PWV, which is widely accepted as the gold standard for assessing arterial stiffness (37). Thirdly, while the eGFR based on creatinine is commonly used in large-scale studies, it may be influenced by non-GFR determinants and may not be completely accurate in assessing kidney function. To ensure the robustness of our findings, we additionally conducted the sensitivity analysis using eGFR calculated by the Modification of Diet in Renal Disease equation and observed the consistent result. Lastly, the performance of PRS may vary across ancestries, even within populations of same ethnic background but from different countries (38). Further evaluation of our findings in other East Asian ancestry other than Chinese is needed.

In conclusion, the present study highlights significant role of baPWV in lower eGFR and increased CKD presence, which seemingly was not influenced by genetic susceptibility. Our findings suggest that arterial stiffness may represent a modifiable risk factor for CKD. Overall, results offer new understandings into how genetic and baPWV mutually contribute to CKD etiology, warranting further research for validation and identification of mechanistic insights.

## Data Availability

The raw data supporting the conclusions of this article will be made available by the authors, without undue reservation.
