# Supplementary material for: Joint effects of arterial stiffness and polygenic risk on kidney function: the Dongfeng-Tongji cohort
Source: Front Cardiovasc Med. 2026 Mar 13;13:1717046. doi: 10.3389/fcvm.2026.1717046 (PMC13021437; doi:10.3389/fcvm.2026.1717046)
Supplement: Supplementary file 1 [file Datasheet1.docx]

**Title:**

Joint effects of arterial stiffness and polygenic risk on kidney function: the Dongfeng-Tongji cohort

**Short title:**

Arterial stiffness, polygenic risk, and kidney function

**Authors:**

Haiqing Zhang, PhD ^a,b†^; Rui Zhang, PhD ^a†^; Yaling He, PhD ^a^; Xuefeng Lai, PhD ^a^; Rong Peng, PhD ^a^; Miao Liu, PhD ^a^; Biao Zhang, PhD ^a^; Huihui Wang, PhD ^c^; Xingjie Hao, PhD ^c^; Liangle Yang, PhD ^a^; Xiaomin Zhang, PhD ^a*^

**Authors affiliations:**

^a^ Department of Occupational and Environmental Health, Ministry of Education Key Laboratory of Environment and Health, and State Key Laboratory of Environmental Health (Incubating), School of Public Health, School of Tongji Medical College, Huazhong University of Science and Technology, Wuhan, Hubei, China.

^b^ Henan Provincial Modern Hospital Management Research Center, Henan Provincial People’s Hospital, People’s Hospital of Zhengzhou University, Zhengzhou, Henan, China.

^c^ Department of Epidemiology and Biostatistics, School of Public Health, School of Tongji Medical College, Huazhong University of Science and Technology, Wuhan, Hubei, China.

† Haiqing Zhang and Rui Zhang contributed equally to this work.

**Supplemental Material**

**Contents:**

Figure S1 Flow chart of study participants in the present study

Figure S2 Multivariable adjusted spline curves for association between baPWV with kidney function

Figure S3 Multivariable adjusted spline curves for association between PRS with kidney function

Figure S4 Interactive effects of PRS and baPWV on kidney function

Table S1 Single nucleotide polymorphism selected for the construction of the PRS

Table S2 Participant characteristics of study population by PRS

Table S3 The joint association of PRS and baPWV with kidney function

Table S4 Sensitivity analysis of associations between baPWV and kidney function

Table S5 Sensitivity analysis of joint association of PRS and baPWV with kidney function

Participants with arterial stiffness and kidney function data in 2018

(N = 21,801)

Participants with kidney function data in 2018 (N = 31,046)

Participants with arterial stiffness data in 2018 (N = 22,593)

Participants in the Dongfeng-Tongji cohort study in 2018 (N = 36,215)

Participants with genotype data

(N = 13,494)

8,307 excluded because of missing genotype information

**Figure S1 Flow chart of study participants in the present study**


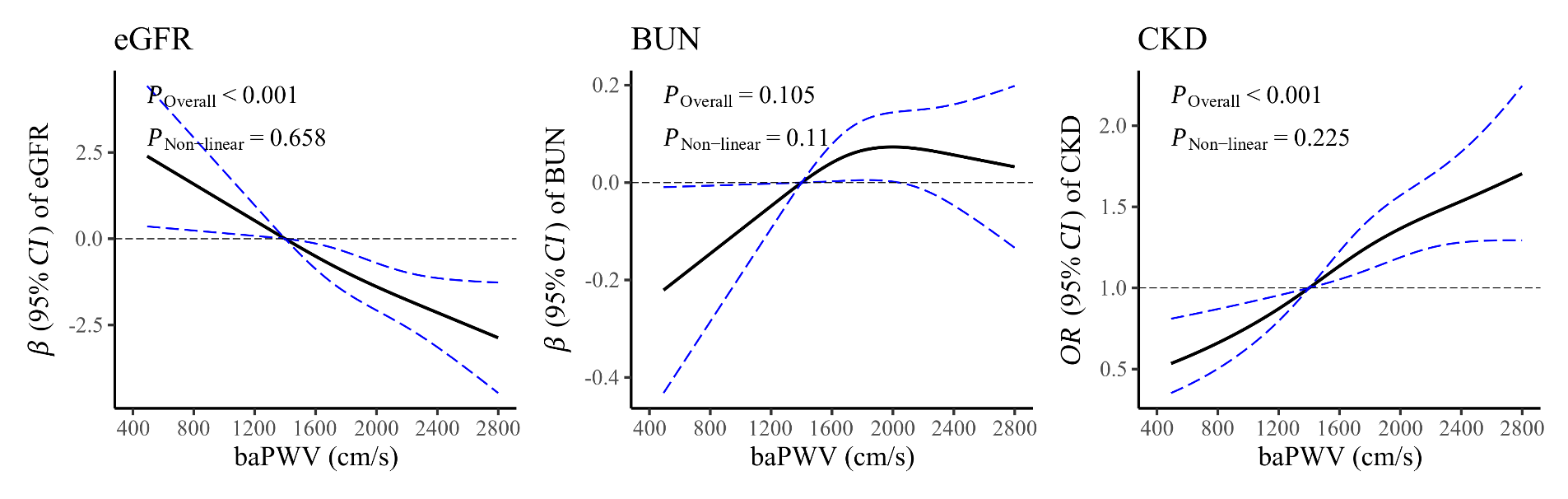


**Figure S2 Multivariable adjusted spline curves for association between baPWV with kidney function**

Adjusted for age, gender, marital status, education, smoking status, drinking status, physical activity, body mass index, fasting plasma glucose, cardiovascular disease, cancer, systolic blood pressure, triglyceride, total cholesterol, and high density lipoprotein cholesterol.

Abbreviations: baPWV, brachial-ankle pulse wave velocity; BUN, blood urea nitrogen; CI, confidence interval; CKD, chronic kidney disease; eGFR, estimated glomerular filtration; OR, odds ratio.

**
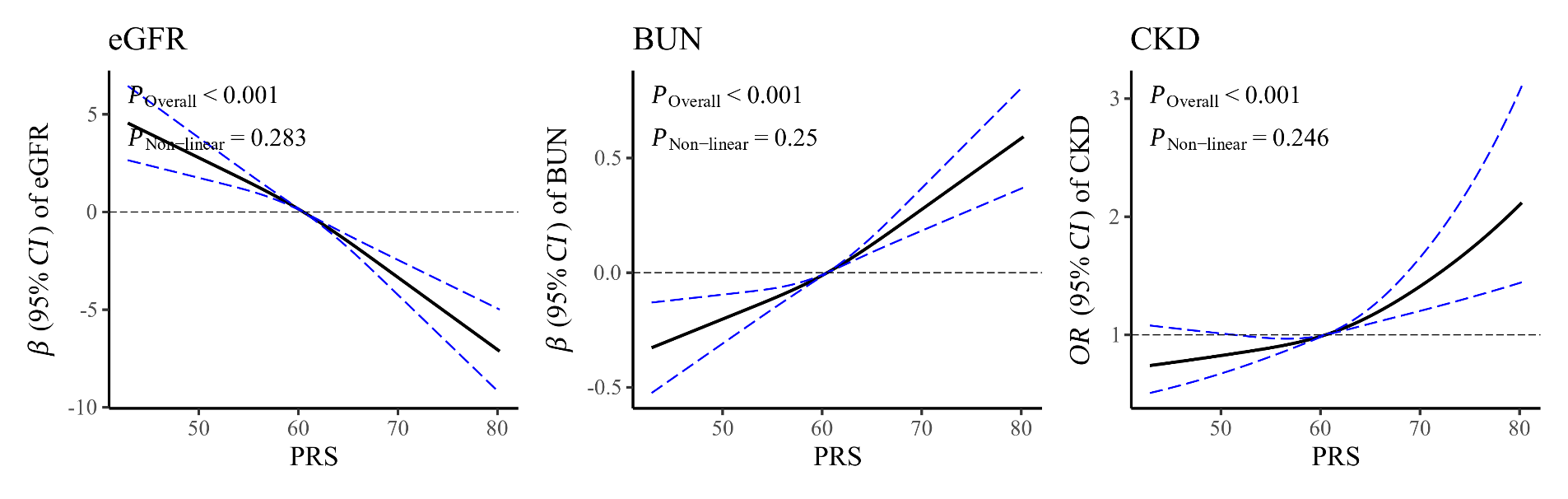
**

**Figure S3 Multivariable adjusted spline curves for association between PRS with kidney function**

Adjusted for age, gender, and the first ten principal components.

Abbreviations: baPWV, brachial-ankle pulse wave velocity; BUN, blood urea nitrogen; CI, confidence interval; CKD, chronic kidney disease; eGFR, estimated glomerular filtration; OR, odds ratio; PGS polygenetic score.


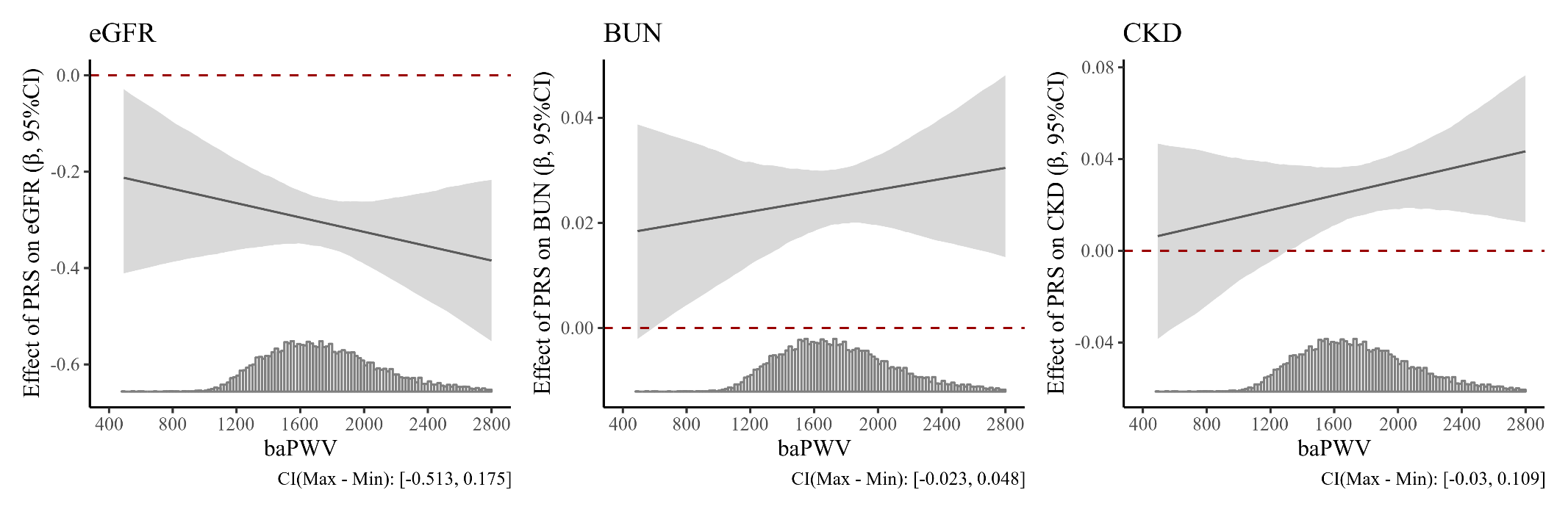


**Figure S4 Interactive effects of PRS and baPWV on kidney function**

Adjusted for age, gender, marital status, education, smoking status, drinking status, physical activity, body mass index, fasting plasma glucose, cardiovascular disease, cancer, systolic blood pressure, triglyceride, total cholesterol, and high density lipoprotein cholesterol.

The histogram of x-axis represented the number of individuals exposed to the corresponding baPWV threshold.

An interval including 0 indicates no statistical difference.

Abbreviations: baPWV, brachial-ankle pulse wave velocity; BUN, blood urea nitrogen; CI, confidence interval; CKD, chronic kidney disease; eGFR, estimated glomerular filtration; PRS, polygenetic score.

**Table S1 Single nucleotide polymorphism selected for the construction of the PRS**

| **Number** | **rsID** | **Chr:BP (GRCh38)** | **Genes** | **Alleles^a^** | **MAF** | **β** | **P** |
| --- | --- | --- | --- | --- | --- | --- | --- |
| 1 | rs2990246 | 1:155227811 | *GBAP1* | G/C | 0.845 | 0.037 | 7.99E-11 |
| 2 | rs848302 | 1:15989624 | *ZBTB17, C1orf64* | T/A | 0.690 | 0.029 | 1.27E-12 |
| 3 | rs34720381 | 1:171486183 | *PRRC2C* | C/T | 0.069 | -0.045 | 1.46E-09 |
| 4 | rs4525087 | 1:23365736 | *ZNF436* | A/C | 0.601 | -0.027 | 5.21E-12 |
| 5 | rs11123169 | 2:113209498 | *PSD4, PAX8* | C/T | 0.732 | 0.033 | 2.38E-15 |
| 6 | rs36096257 | 2:121281207 | *TFCP2L1* | G/A | 0.052 | -0.063 | 2.24E-13 |
| 7 | rs3788967 | 2:162241460 | *FAP* | G/A | 0.130 | 0.030 | 4.75E-08 |
| 8 | rs16856823 | 2:169343942 | *LRP2* | A/T | 0.193 | 0.047 | 4.68E-22 |
| 9 | rs72944180 | 2:210698200 | *CPS1, ERBB4* | C/T | 0.156 | -0.040 | 5.31E-15 |
| 10 | rs10189682 | 2:216781832 | *IGFBP5, TNP1* | G/A | 0.446 | 0.023 | 1.31E-09 |
| 11 | rs1260326 | 2:27508073 | *GCKR* | T/C | 0.441 | -0.032 | 3.59E-17 |
| 12 | rs307558 | 3:12053630 | *SYN2* | G/A | 0.744 | 0.027 | 1.20E-09 |
| 13 | rs2332050 | 3:121916971 | *SLC15A2* | C/G | 0.732 | -0.025 | 1.58E-08 |
| 14 | rs16853722 | 3:169432844 | *MECOM* | T/C | 0.279 | -0.024 | 1.67E-08 |
| 15 | rs5029970 | 3:186716702 | *KNG1* | T/C | 0.301 | -0.024 | 5.27E-09 |
| 16 | rs6851943 | 4:100203265 | *LOC101929353* | G/T | 0.459 | -0.022 | 6.13E-09 |
| 17 | rs4690095 | 4:3419582 | *RGS12* | C/T | 0.470 | -0.023 | 1.83E-09 |
| 18 | rs28817415 | 4:76480299 | *SHROOM3* | C/T | 0.217 | -0.050 | 4.98E-28 |
| 19 | rs12509595 | 4:80261400 | *PRDM8, FGF5* | T/C | 0.301 | 0.032 | 1.57E-14 |
| 20 | rs2725261 | 4:88115201 | *ABCG2* | C/T | 0.634 | -0.023 | 8.72E-09 |
| 21 | rs11742501 | 5:151727535 | *CTB-113P19.1, ATOX1* | T/C | 0.094 | 0.036 | 2.48E-08 |
| 22 | rs12659266 | 5:177380196 | *RGS14, SLC34A1* | C/T | 0.251 | -0.035 | 3.05E-16 |
| 23 | rs7714709 | 5:34510282 | *C1QTNF3-AMACR, RAI14* | G/A | 0.729 | 0.030 | 9.27E-12 |
| 24 | rs6420041 | 5:3595864 | *IRX1* | G/A | 0.480 | 0.023 | 2.87E-08 |
| 25 | rs241812 | 6:100443115 | *SIM1* | A/G | 0.571 | 0.023 | 1.57E-09 |
| 26 | rs2781656 | 6:131561431 | *AKAP7, ARG1* | C/T | 0.320 | -0.028 | 2.57E-12 |
| 27 | rs316020 | 6:160248049 | *SLC22A2* | A/G | 0.949 | -0.057 | 7.32E-12 |
| 28 | rs117463603 | 6:32612725 | *HLA-DRB1, HLA-DQA1* | G/A | 0.140 | 0.038 | 1.55E-09 |
| 29 | rs9368805 | 6:34257298 | *C6orf1, RPS10-NUDT3* | T/C | 0.773 | 0.029 | 1.67E-09 |
| 30 | rs881858 | 6:43838872 | *VEGFA, LINC01512* | G/A | 0.879 | -0.032 | 3.46E-08 |
| 31 | rs4715491 | 6:54899522 | *FAM83B* | A/G | 0.229 | -0.034 | 1.13E-13 |
| 32 | rs6907843 | 6:89399655 | *RRAGD* | C/T | 0.084 | -0.041 | 2.86E-09 |
| 33 | rs1533988 | 7:1253374 | *UNCX, MICALL2* | A/T | 0.333 | -0.060 | 8.68E-39 |
| 34 | rs75834729 | 8:129359392 | *CCDC26* | C/T | 0.290 | 0.026 | 2.45E-10 |
| 35 | rs1705694 | 8:23912105 | *STC1, ADAM28* | A/G | 0.836 | 0.036 | 1.17E-12 |
| 36 | rs75174967 | 10:102517336 | *SUFU* | G/A | 0.152 | -0.032 | 1.58E-09 |
| 37 | rs7475348 | 10:68205420 | *MYPN* | C/T | 0.315 | 0.034 | 2.99E-16 |
| 38 | rs10840341 | 11:2095263 | *H19, IGF2* | T/A | 0.529 | -0.021 | 4.66E-08 |
| 39 | rs963837 | 11:30727543 | *MPPED2, DCDC5* | T/C | 0.343 | 0.041 | 7.93E-22 |
| 40 | rs11039297 | 11:47559891 | *CELF1, PTPMT1* | G/A | 0.250 | -0.028 | 3.38E-10 |
| 41 | rs691329 | 11:50428834 | *LOC646813, OR4A5* | C/G | 0.666 | 0.022 | 2.93E-08 |
| 42 | rs12806498 | 11:55304706 | *TRIM51HP, OR4A16* | A/T | 0.321 | -0.024 | 6.90E-09 |
| 43 | rs4945275 | 11:78408776 | *GAB2* | G/A | 0.478 | 0.021 | 1.47E-08 |
| 44 | rs10850813 | 12:109821615 | *TRPV4* | C/T | 0.680 | -0.026 | 4.90E-09 |
| 45 | rs79105258 | 12:111280427 | *CUX2* | C/A | 0.250 | -0.045 | 3.88E-22 |
| 46 | rs11062070 | 12:234466 | *SLC6A13* | C/T | 0.401 | -0.024 | 2.82E-09 |
| 47 | rs4399402 | 12:23746659 | *SOX5* | A/G | 0.818 | 0.028 | 6.78E-09 |
| 48 | rs3782787 | 12:3249095 | *TSPAN9* | C/G | 0.257 | -0.026 | 6.80E-10 |
| 49 | rs1275609 | 12:75877403 | *KRR1, PHLDA1* | G/A | 0.594 | 0.028 | 1.33E-11 |
| 50 | rs1536622 | 13:110412179 | *COL4A2* | T/C | 0.678 | 0.023 | 1.20E-08 |
| 51 | rs67332916 | 13:42175575 | *DGKH* | C/T | 0.398 | 0.027 | 2.95E-11 |
| 52 | rs626277 | 13:71773564 | *DACH1* | A/C | 0.850 | 0.038 | 4.41E-13 |
| 53 | rs8023655 | 15:50833122 | *SPPL2A, AP4E1* | T/C | 0.207 | 0.027 | 8.67E-09 |
| 54 | rs10518732 | 15:53646845 | *WDR72* | G/C | 0.392 | 0.048 | 4.82E-37 |
| 55 | rs28607641 | 15:75899303 | *UBE2Q2* | A/T | 0.503 | -0.029 | 3.60E-11 |
| 56 | rs79170539 | 15:80844377 | *CEMIP* | A/T | 0.233 | 0.027 | 1.19E-08 |
| 57 | rs4966025 | 15:98766313 | *IGF1R* | G/A | 0.489 | 0.025 | 1.78E-10 |
| 58 | rs77924615 | 16:20381010 | *PDILT* | G/A | 0.219 | 0.073 | 5.81E-59 |
| 59 | rs12935539 | 16:51721080 | *SALL1, LINC01571* | T/C | 0.315 | -0.024 | 3.86E-09 |
| 60 | rs7212715 | 17:39451651 | *MED1* | C/T | 0.747 | -0.038 | 4.32E-14 |
| 61 | rs9895661 | 17:61379228 | *BCAS3* | C/T | 0.478 | 0.048 | 1.08E-28 |
| 62 | rs16942751 | 18:26813249 | *PCAT18, AQP4* | C/A | 0.296 | -0.024 | 6.05E-09 |
| 63 | rs2337106 | 18:48934533 | *SMAD7* | C/G | 0.533 | 0.022 | 1.18E-08 |
| 64 | rs549752 | 18:79398225 | *NFATC1* | A/G | 0.682 | 0.042 | 8.96E-25 |
| 65 | rs6026578 | 20:58888417 | *LOC101927932* | C/G | 0.723 | 0.027 | 1.41E-10 |
| 66 | rs128494 | 21:36461960 | *CLDN14* | T/C | 0.429 | 0.023 | 8.59E-10 |
| 67 | rs6001939 | 22:40496790 | *MKL1* | C/T | 0.266 | 0.033 | 6.85E-13 |
| 68 | rs5987107 | X:153610129 | *LOC105373383, DUSP9* | A/G | 0.675 | 0.023 | 9.69E-12 |
| 69 | rs12845465 | X:8944553 | *FAM9A, FAM9B* | T/C | 0.271 | -0.021 | 4.91E-09 |

^a^ Alleles present reference allele/risk allele.

Abbreviation: BP: base position; BUN, blood urea nitrogen; Cr, creatinine; eGFR, estimated glomerular filtration; MAF, minor allele frequency; PGS, polygenetic score.

**Table S2 Participant characteristics of study population by PRS**

| **Characteristics** | **PRS** | | |  |
| --- | --- | --- | --- | --- |
|  | **Low** | **Medium** | **High** | ***P*** |
| No. of participants, n (%) | 2,698 | 8,097 | 2,699 |  |
| Age (year), mean ± SD | 68.3±8.1 | 68.3±8.0 | 68.3±7.9 | 0.866 |
| Gender, n (%) |  |  |  | 0.339 |
| Male | 1,133 (42.0) | 3,305 (40.8) | 1,081 (40.1) |  |
| Female | 1,565 (58.0) | 4,792 (59.2) | 1,618 (59.9) |  |
| Education, n (%) |  |  |  | 0.557 |
| Primary school or below | 644 (23.9) | 1,843 (22.8) | 614 (22.7) |  |
| Junior or senior high school | 1,819 (67.4) | 5,552 (68.6) | 1,869 (69.2) |  |
| College or above | 235 (8.7) | 702 (8.7) | 216 (8.0) |  |
| Marital status, n (%) |  |  |  | 0.070 |
| Unmarried/divorced/widowed | 356 (13.2) | 1,135 (14.0) | 414 (15.3) |  |
| Married/remarried | 2,342 (86.8) | 6,962 (86.0) | 2,284 (84.7) |  |
| Current smoker, n (%) | 345 (12.8) | 898 (11.1) | 302 (11.2) | 0.050 |
| Current drinker, n (%) | 552 (20.5) | 1516 (18.7) | 467 (17.3) | 0.012 |
| Physical activity, n (%) | 2,467 (91.5) | 7,440 (91.9) | 2,443 (90.5) | 0.082 |
| BMI (kg/m^2^), mean ± SD | 24.7±3.3 | 24.7±3.4 | 24.7±3.4 | 0.658 |
| SBP (mmHg), mean ± SD | 139.3±21.3 | 138.8±21.1 | 138.4±20.9 | 0.223 |
| FPG (mmol/L), mean ± SD | 6.0±1.9 | 6.0±1.9 | 6.0±1.9 | 0.710 |
| TG (mmol/L), mean ± SD | 1.5±1.0 | 1.5±1.0 | 1.5±1.0 | 0.946 |
| TC (mmol/L), mean ± SD | 4.8±1.0 | 4.8±1.1 | 4.8±1.1 | 0.773 |
| HDL-C (mmol/L), mean ± SD | 1.4±0.4 | 1.4±0.4 | 1.4±0.4 | 0.874 |
| Cr (μmol/L), mean ± SD | 74.4±29.3 | 75.8±26.2 | 78.3±24.0 | <0.001 |
| eGFR (ml/min/1.73 m^2^), mean ± SD | 81.5±16.9 | 80.0±16.7 | 77.6±16.7 | <0.001 |
| BUN (mmol/L), mean ± SD | 5.3±1.6 | 5.5±1.6 | 5.7±1.7 | <0.001 |
| CKD, n (%) | 354 (13.1) | 1129 (13.9) | 456 (16.9) | <0.001 |

The PRS was classified as low (the bottom quintile), medium (the second to the fourth quintile), and high (the top quintile).

Abbreviation: baPWV, brachial-ankle pulse wave velocity; BMI, body mass index; BUN, blood urea nitrogen; CKD, chronic kidney disease; Cr, creatinine; eGFR, estimated glomerular filtration; FPG, fasting plasma glucose; HDL-C, high density lipoprotein cholesterol; PGS, polygenetic score; SBP, systolic blood pressure; SD, standard deviation; TC, total cholesterol, and TG, triglyceride.

**Table S3 The joint association of PRS and baPWV with kidney function**

| **PRS** |  | **baPWV (cm/s)** |  |
| --- | --- | --- | --- |
|  | **< 1400** | **1400 -< 1800** | **≥ 1800** |
| **β (95% CI) of eGFR** | | | |
| Low | 0 (ref) | 0.049 (-1.723, 1.821) | -0.893 (-2.724, 0.939) |
| Medium | -1.287 (-2.995, 0.421) | -1.350 (-2.948, 0.247) | -2.845 (-4.517, -1.173) |
| High | -2.143 (-4.239, -0.047) | -4.355 (-6.112, -2.597) | -5.317 (-7.161, -3.472) |
| **β (95% CI) of BUN** | | | |
| Low | 0 (ref) | 0.023 (-0.160, 0.206) | 0.020 (-0.169, 0.209) |
| Medium | 0.132 (-0.045, 0.308) | 0.160 (-0.005, 0.325) | 0.218 (0.045, 0.391) |
| High | 0.233 (0.016, 0.449) | 0.374 (0.192, 0.556) | 0.369 (0.178, 0.560) |
| **OR (95% CI) of CKD** | | | |
| Low | 1 (ref) | 1.05 (0.72-1.55) | 1.09 (0.74-1.61) |
| Medium | 0.92 (0.62-1.36) | 1.00 (0.70-1.43) | 1.38 (0.96-1.98) |
| High | 1.22 (0.77-1.93) | 1.31 (0.90-1.91) | 1.74 (1.19-2.55) |

Adjusted for age, gender, marital status, education, smoking status, drinking status, physical activity, body mass index, fasting plasma glucose, cardiovascular disease, cancer, systolic blood pressure, triglyceride, total cholesterol, and high density lipoprotein cholesterol.

The PRS was classified as low (the bottom quintile), medium (the second to the fourth quintile), and high (the top quintile).

Abbreviations: baPWV, brachial-ankle pulse wave velocity; BUN, blood urea nitrogen; Cr, creatinine; eGFR, estimated glomerular filtration.

Table S4 Sensitivity analysis of associations between baPWV and kidney function

| **Models** | **baPWV (cm/s)** | | | | |
| --- | --- | --- | --- | --- | --- |
|  | **< 1400** | **1400 -< 1800** | **≥ 1800** | ***P* _trend_** | **Per 100 cm/s increase in baPWV** |
| **β (95% CI) of eGFR** | | | | | |
| Model 1 | 0 (ref) | -0.451 (-1.275, 0.373) | **-1.682 (-2.634, -0.731)** | <0.001 | **-0.204 (-0.292, -0.117)** |
| Model 2 | 0 (ref) | -0.476 (-1.343, 0.391) | **-1.538 (-2.538, -0.538)** | <0.001 | **-0.183 (-0.275, -0.091)** |
| Model 3 | 0 (ref) | -0.765 (-1.718, 0.188) | **-1.929 (-3.074, -0.784)** | <0.001 | **-0.220 (-0.331, -0.110)** |
| Model 4 | 0 (ref) | -0.486 (-1.831, 0.859) | **-1.925 (-3.477, -0.373)** | 0.001 | **-0.249 (-0.392, -0.106)** |
| **β (95% CI) of BUN** | | | | | |
| Model 1 | 0 (ref) | 0.043 (-0.040, 0.125) | 0.067 (-0.029, 0.162) | 0.603 | 0.002 (-0.006, 0.011) |
| Model 2 | 0 (ref) | 0.047 (-0.042, 0.136) | 0.053 (-0.050, 0.155) | 0.930 | 0.000 (-0.009, 0.010) |
| Model 3 | 0 (ref) | 0.072 (-0.022, 0.167) | **0.119 (0.005, 0.233)** | 0.354 | 0.005 (-0.006, 0.016) |
| **OR (95% CI) of CKD** | | | | | |
| Model 1 | 1 (ref) | 1.07 (0.90-1.28) | **1.38 (1.14-1.68)** | <0.001 | **1.04 (1.02-1.05)** |
| Model 2 | 1 (ref) | 1.06 (0.88-1.28) | **1.35 (1.11-1.66)** | <0.001 | **1.03 (1.02-1.05)** |
| Model 3 | 1 (ref) | **1.27 (1.02-1.58)** | **1.64 (1.29-2.10)** | <0.001 | **1.05 (1.03-1.07)** |
| Model 4 | 1 (ref) | 1.09 (0.91-1.30) | **1.36 (1.11-1.65)** | <0.001 | **1.04 (1.02-1.06)** |

All models were adjusted for age, gender, marital status, education, smoking status, drinking status, physical activity, body mass index, fasting plasma glucose, cardiovascular disease, cancer, systolic blood pressure, triglyceride, total cholesterol, and high density lipoprotein cholesterol.

Model 1 was adjusted for covariates above excluding population with eGFR under 15 ml/min/1.73 m^2^.

Model 2 was adjusted for covariates above excluding population with cancer.

Model 3 was adjusted for covariates above excluding population with CVD.

Model 4 was adjusted for covariates above and calculated the eGFR using the Modification of Diet in Renal Disease equation.

The PRS was classified as low (the bottom quintile), medium (the second to the fourth quintile), and high (the top quintile).

Bold values denote statistically significant.

Abbreviations: baPWV, brachial-ankle pulse wave velocity; BUN, blood urea nitrogen; CI, confidence interval; CKD, chronic kidney disease; eGFR, estimated glomerular filtration; OR, odds ratio.

Table S5 Sensitivity analysis of joint association of PRS and baPWV with kidney function

| **PRS** | **Low** | | | **Medium** | | | **High** | | |
| --- | --- | --- | --- | --- | --- | --- | --- | --- | --- |
| **baPWV** | **< 1400** | **1400 -< 1800** | **≥ 1800** | **< 1400** | **1400 -< 1800** | **≥ 1800** | **< 1400** | **1400 -< 1800** | **≥ 1800** |
| **β (95% CI) of eGFR** | | | | | | | | | |
| Model 1 | 0 (ref) | 0.235 (-1.519, 1.989) | -0.858 (-2.670, 0.954) | -1.283 (-2.972, 0.407) | -1.300 (-2.880, 0.280) | -2.763 (-4.418, -1.109) | -2.139 (-4.212, -0.065) | -4.366 (-6.104, -2.627) | -5.219 (-7.045, -3.393) |
| Model 2 | 0 (ref) | 0.085 (-1.763, 1.934) | -0.693 (-2.601, 1.216) | -1.280 (-3.064, 0.504) | -1.439 (-3.108, 0.230) | -2.799 (-4.545, -1.053) | -2.625 (-4.814, -0.437) | -4.443 (-6.280, -2.606) | -5.000 (-6.922, -3.078) |
| Model 3 | 0 (ref) | -0.686 (-2.732, 1.360) | -1.977 (-4.157, 0.202) | -2.029 (-3.955, -0.103) | -2.371 (-4.197, -0.546) | -3.650 (-5.595, -1.705) | -3.363 (-5.696, -1.030) | -5.264 (-7.281, -3.248) | -6.210 (-8.389, -4.032) |
| Model 4 | 0 (ref) | 0.932 (-1.931, 3.794) | 0.159 (-2.798, 3.117) | -1.470 (-4.229, 1.289) | -1.592 (-4.172, 0.987) | -3.353 (-6.054, -0.652) | -3.290 (-6.676, 0.096) | -5.948 (-8.787, -3.110) | -7.378 (-10.358, -4.398) |
| **β (95% CI) of BUN** | | | | | | | | | |
| Model 1 | 0 (ref) | -0.015 (-0.191, 0.161) | 0.014 (-0.167, 0.196) | 0.131 (-0.038, 0.301) | 0.153 (-0.005, 0.312) | 0.201 (0.035, 0.367) | 0.232 (0.024, 0.441) | 0.376 (0.201, 0.550) | 0.342 (0.158, 0.525) |
| Model 2 | 0 (ref) | 0.046 (-0.144, 0.236) | -0.005 (-0.201, 0.191) | 0.130 (-0.053, 0.314) | 0.155 (-0.016, 0.327) | 0.195 (0.016, 0.375) | 0.260 (0.035, 0.485) | 0.362 (0.173, 0.551) | 0.331 (0.133, 0.528) |
| Model 3 | 0 (ref) | 0.052 (-0.152, 0.256) | 0.083 (-0.134, 0.300) | 0.122 (-0.070, 0.313) | 0.195 (0.013, 0.377) | 0.259 (0.066, 0.453) | 0.283 (0.051, 0.515) | 0.360 (0.159, 0.560) | 0.384 (0.167, 0.601) |
| **OR (95% CI) of CKD** | | | | | | | | | |
| Model 1 | 1 (ref) | 1.03 (0.70-1.52) | 1.08 (0.74-1.60) | 0.92 (0.62-1.36) | 0.99 (0.70-1.42) | 1.37 (0.95-1.96) | 1.22 (0.77-1.93) | 1.31 (0.90-1.91) | **1.73 (1.18-2.53)** |
| Model 2 | 1 (ref) | 1.01 (0.67-1.51) | 1.02 (0.68-1.53) | 0.90 (0.60-1.35) | 0.99 (0.69-1.44) | 1.36 (0.93-1.98) | 1.27 (0.79-2.05) | 1.28 (0.86-1.90) | **1.63 (1.10-2.43)** |
| Model 3 | 1 (ref) | **1.75 (1.03-2.97)** | **1.74 (1.01-2.99)** | 1.33 (0.79-2.24) | 1.60 (0.98-2.62) | **2.25 (1.36-3.72)** | **1.83 (1.02-3.29)** | **2.07 (1.24-3.47)** | **2.75 (1.62-4.66)** |
| Model 4 | 1 (ref) | 1.11 (0.75-1.64) | 1.15 (0.77-1.70) | 0.94 (0.64-1.39) | 1.01 (0.70-1.44) | 1.40 (0.97-2.02) | 1.27 (0.81-2.01) | 1.41 (0.96-2.06) | **1.55 (1.05-2.30)** |

All models were adjusted for age, gender, marital status, education, smoking status, drinking status, physical activity, body mass index, fasting plasma glucose, cardiovascular disease, cancer, systolic blood pressure, triglyceride, total cholesterol, and high density lipoprotein cholesterol.

Model 1 was adjusted for covariates above excluding population with eGFR under 15 ml/min/1.73 m^2^.

Model 2 was adjusted for covariates above excluding population with cancer.

Model 3 was adjusted for covariates above excluding population with CVD.

Model 4 was adjusted for covariates above and calculated the eGFR using the Modification of Diet in Renal Disease equation.

The PRS was classified as low (the bottom quintile), medium (the second to the fourth quintile), and high (the top quintile).

Bold values denote statistically significant.

Abbreviations: baPWV, brachial-ankle pulse wave velocity; BUN, blood urea nitrogen; CI, confidence interval; CKD, chronic kidney disease; eGFR, estimated glomerular filtration; OR, odds ratio; PGS, polygenetic score
